# Twelve years' experience of the mini-Bankart repair for recurrent anterior dislocation of the shoulder

**DOI:** 10.4103/0973-6042.57936

**Published:** 2009

**Authors:** Alan Cooney, Satyajit Sinha, Alexander Craig Campbell

**Affiliations:** Department of Orthopaedic Surgery, Monklands Hospital, Airdrie, UK

**Keywords:** Mini-Bankart repair, recurrent anterior shoulder dislocation, shoulder surgery

## Abstract

Stabilization for recurrent anterior shoulder dislocation can be achieved through either an open or arthroscopic approach. The former tends to have a lower recurrence rate but longer rehabilitation.The technique of mini-Bankart repair has been used at this establishment since 1996. We retrospectively reviewed the patients that had undergone this procedure. We describe our experience of the mini-Bankart procedure and the results in 24 patients with a mean follow-up of 56 months (range, 12-144 months).

The technique is a direct mini-approach to the shoulder joint, preserving the inferior portion of subscapularis. Where present, a Bankart lesion is repaired with two GII Mitek anchors (Ethicon) and the capsule reefed. There were no incidences of repeat anterior dislocation, and the average time period taken to return to work was 8.8 weeks. We recommend this technique due to its low recurrence rate and satisfactory return to normal function.

## INTRODUCTION

Young adults that sustain a traumatic dislocation of the shoulder have a high risk of further dislocations. The reason for over 85% of these recurrent dislocations is the Bankart lesion[1] (named after Blundell Bankart, who first described it), whereby the inferior half of the anterior glenoid labrum becomes detached.[2]

The indication for operative stabilization via a Bankart repair is recurrent dislocations with unidirectional instability. This can be achieved through either an open or arthroscopic approach. In the classic open Bankart procedure, the subscapularis tendon is reflected medially, thereby exposing the anterior labrum and allowing surgical repair.[3]

It is well recognized that the open approach yields consistently low rates of recurrent instability (5% in a recent meta-analysis),[4] although there have been some reported incidents of postoperative subscapularis rupture.[5,6]

Surgeons favoring arthroscopic repair describe benefits relating to smaller incisions, an improved range of postoperative movement, an earlier return to work and sport and a lower risk of subscapularis failure. It has been demonstrated that patients treated arthroscopically do have higher functional scores postoperatively, but there is a significantly increased risk of subsequent instability and re-dislocation when compared to the open technique (12-16%).[4]

Consequently, the senior author (ACC) has developed a mini-open Bankart procedure, with the aim of combining the stability of the open approach with the improved functional outcomes of the arthroscopic technique. We describe the procedure here and the postoperative outcome in 24 patients with a mean follow-up of 56 months (range, 12-144 months).

## TECHNIQUE

**Figure 1:**
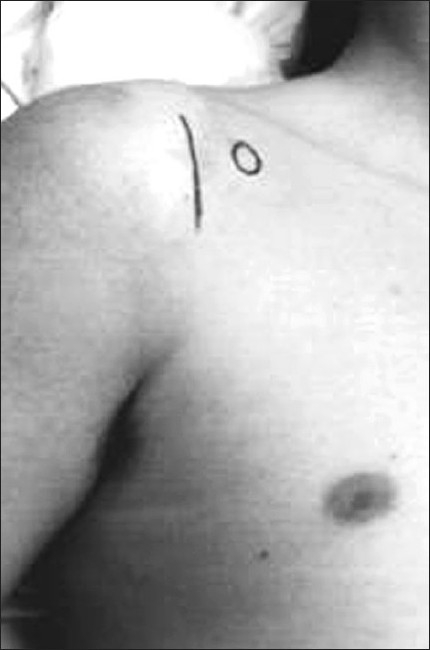
Incision landmarks

**Figure 2:**
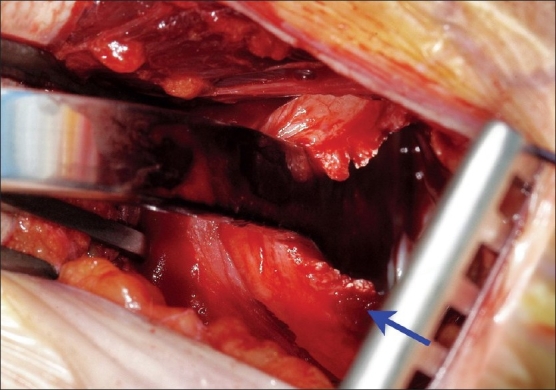
Per-operative image with arrow indicating the preserved inferior third of the subscapularis muscle

The patient is placed in the deck chair position. A 5-cm vertical incision is made 2 cm lateral to the coracoid process, extending towards the axillary fold [Figure 1]. The conjoint tendon arising from the coracoid process is identified and retracted medially, exposing the subscapularis. In the classical open approach, the entire subscapularis tendon is then divided and reflected medially.[3] In the mini-Bankart approach, only the superior two thirds of the subscapularis tendon is divided, 1 to 2 cm from its insertion, preserving the inferior third [Figure 2]. The capsule is subsequently divided, leaving a cuff to close and exposing the gleno-humeral joint.

**Figure 3:**
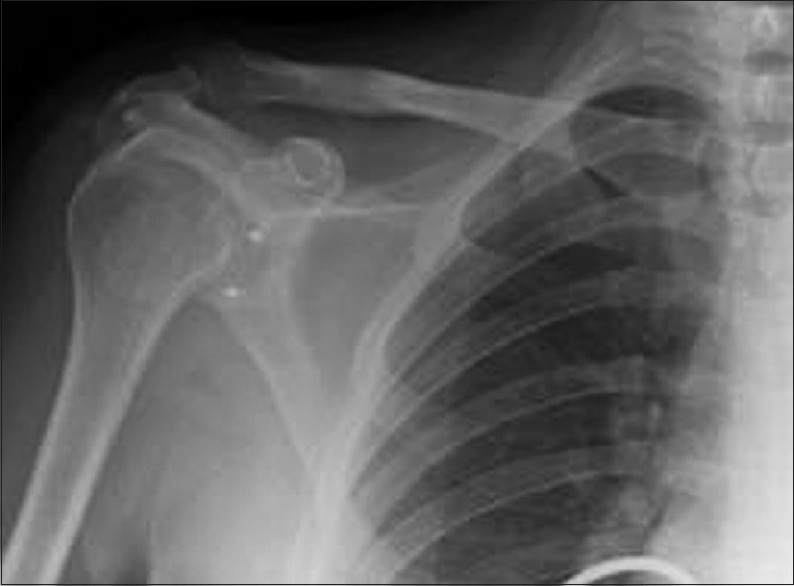
Placement of Mitek anchors

**Figure 4:**
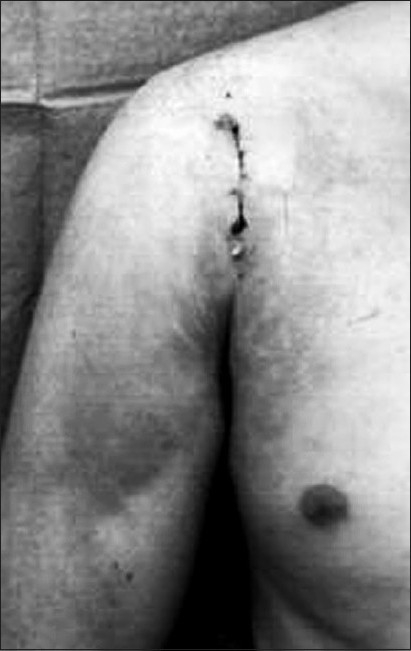
Wound scar

Where present, a Bankart lesion is freshened with a burr drill and repaired with two GII Mitek anchors (Ethicon, Livingston, UK). The anchors are placed at 3- and 5-o'clock positions for right shoulder [Figure 3]; and 7- and 9-o'clock positions for left shoulder, passing the sutures through the capsule. None of the patients had associated glenoid bone loss. The capsule itself is then closed with polydioxanone (PDS) II (Ethicon). Two Ethibond (Ethicon) sutures are tied over outside the capsule and then to each other, closing the Bankart lesion. The subscapularis tendon is repaired with PDS. Subsequent closure is in layers with a subcuticular suture for the skin [Figure 4].

Postoperatively, patients are placed in a polysling for 6 weeks; ensuring external rotation is avoided for the initial 3 weeks. Patients are under immediate supervision of physiotherapists.

## RESULTS

Twenty-four procedures have been performed in 24 patients (21 males, 3 females) with a mean age of 28 years (range, 19-42 years) and a mean follow-up of 56 months (range, 12-144 months). There were 10 right-sided and 14 left-sided procedures. Twenty-one primary stabilizations were performed; with 3 revision procedures, all of whom had undergone their primary surgery in other institutions. All 24 patients had recurrent dislocations with unidirectional instability, and all had a Bankart lesion evident at surgical repair.

There were no incidences of repeat anterior dislocation, although there was 1 posterior dislocation 2 years postoperatively following subsequent traumatic event, which was managed conservatively. Twenty-two patients have returned to work after a mean duration of 8.8 weeks (range, 4-16 weeks). Those in desk jobs returned after 5.6 weeks, while manual workers recommenced work after 11.2 weeks (range, 7-16 weeks). Of the 2 patients not returning to work, one patient had been unemployed prior to developing shoulder instability and the other is awaiting stabilization of the contralateral shoulder. All patients have also returned to sport, after a mean time period of 22 weeks (range, 12-52 weeks) postoperatively. One patient complained of perceived limitation of movement; there was 1 incident of superficial wound dehiscence and 3 incidents of dysesthesia at the scar. There were no episodes of subscapularis tendon rupture.

## DISCUSSION

Recurrent shoulder instability is a relatively common condition usually affecting young adults. Detachment of the antero-inferior labrum (the Bankart lesion) facilitates recurrent anterior instability and can be repaired via an open or arthroscopic approach.

Based upon the current literature, open stabilization remains the gold standard, with a statistically significant lower risk of subsequent instability (8% *vs*. 18%) and lower re-dislocation rates (5% *vs*. 12%) when compared to arthroscopic approach. The meta-analysis by Lenters *et al*. comparing open and arthroscopic repairs also demonstrated that the open repair is more successful in enabling patients to return to their previous work and/or sport.[4]

However, the open approach is not without complications. It has been demonstrated that patients undergoing arthroscopic stabilization have higher Rowe scores and may have an improved range of movement.[4] Furthermore, there are some concerns: Dividing the subscapularis tendon in its entirety (as in the classic open approach) may result in postoperative subscapularis insufficiency;[6] in addition, there have been reported cases of postoperative subscapularis tendon rupture.[5‐6]

In our series, there have been no postoperative anterior dislocations, including the 3 patients that underwent revision surgery. The overall dislocation rate is superior when compared with the current literature regarding the classic open repair (5%) or arthroscopic repair (12%-16%).[4,7]

Return to work and sport in our patient group was also comparable with both open and arthroscopic stabilization.[4,8‐10] All previously employed patients returned to their preoperative job, half of whom were performing manual duties. Also, all patients returned to their previous sports, although with the exception of 1 patient; these were leisure activities. The patient who did not return to the previous sport participated in competitive football, returning at 12 weeks.

Our patient group reported no serious complications. Three have some scar dysaesthesia, although this has previously been recognized in both open and arthroscopic Bankart repairs.[4] One of these patients had a superficial wound dehiscence, subsequently healing by secondary intervention. However, there has been no concern over this individual's range of movement. One patient did feel a subjectively decreased range of postoperative shoulder movement but has returned successfully to work and swimming. We have not encountered any problems with either subscapularis muscle dysfunction or rupture.

However, as the study is retrospective, it was not possible to formally compare functional scores preoperatively and postoperatively. Nevertheless, subjectively, all patients felt that their quality of life had improved and were satisfied with their outcome.

We recommend mini-Bankart repair technique due to its low recurrence rate and satisfactory return to function.
